# Increasing quality and managing complexity in neuroinformatics software development with continuous integration

**DOI:** 10.3389/fninf.2012.00031

**Published:** 2013-01-03

**Authors:** Yury V. Zaytsev, Abigail Morrison

**Affiliations:** ^1^Institute of Neuroscience and Medicine (INM-6), Computational and Systems Neuroscience, Jülich Research CenterJülich, Germany; ^2^Simulation Laboratory Neuroscience – Bernstein Facility for Simulation and Database Technology, Institute for Advanced Simulation, Jülich Research Center, Jülich Aachen Research AllianceJülich, Germany; ^3^Faculty of Biology, Albert-Ludwig University of FreiburgFreiburg im Breisgau, Germany; ^4^Institute of Cognitive Neuroscience, Faculty of Psychology, Ruhr-University BochumBochum, Germany; ^5^Bernstein Center Freiburg, Albert-Ludwig University of FreiburgFreiburg im Breisgau, Germany

**Keywords:** software development, testing, process management, quality control, complexity management

## Abstract

High quality neuroscience research requires accurate, reliable and well maintained neuroinformatics applications. As software projects become larger, offering more functionality and developing a denser web of interdependence between their component parts, we need more sophisticated methods to manage their complexity. If complexity is allowed to get out of hand, either the quality of the software or the speed of development suffer, and in many cases both. To address this issue, here we develop a scalable, low-cost and open source solution for continuous integration (CI), a technique which ensures the quality of changes to the code base during the development procedure, rather than relying on a pre-release integration phase. We demonstrate that a CI-based workflow, due to rapid feedback about code integration problems and tracking of code health measures, enabled substantial increases in productivity for a major neuroinformatics project and additional benefits for three further projects. Beyond the scope of the current study, we identify multiple areas in which CI can be employed to further increase the quality of neuroinformatics projects by improving development practices and incorporating appropriate development tools. Finally, we discuss what measures can be taken to lower the barrier for developers of neuroinformatics applications to adopt this useful technique.

## 1. Introduction

### 1.1. General overview

In the last decades software has become an integral part of any research-related activity. Scientists are using various software packages to acquire, process and analyze experimental data, but also to aid their theoretical investigations, such as to create and simulate computational models. Many employ standard applications designed to be used as a part of specific procedures, some use more generic packages and libraries, such as computer algebra systems, simulation environments, etc. Notably, the number of researchers who are creating their own software packages to address their particular needs, or scripts to integrate several existing systems in a processing pipeline is ever increasing.

Most scientists, however, are not professional software engineers by training, and therefore the latest developments from the software industry often only make it into widespread adoption of the scientific community with a significant delay (Wilson, 2006). This lag used to be viewed as an acceptable compromise in the past, since most scientific software development was focused on specialized solutions or in-house code tailored to narrow classes of applications, often maintained by a single group or laboratory consisting of only a handful of developers. In such situations, the effort of implementing latest advances in software development technologies from the industry is seen as an investment that is just not going to pay off during the lifecycle of the project.

Currently, there is a clear trend toward large-scale collaboration-based projects, such as ITER (http://www.iter.org), LHC by CERN (http://lhc.web.cern.ch/lhc), LIGO (http://www.ligo.org), and many others, also in the field of neurosciences represented by, for instance, the Human Brain Project (http://www.humanbrainproject.eu) and BrainScaleS (http://brainscales.kip.uni-heidelberg.de), or The Boston Retinal Implant Project (http://www.bostonretinalimplant.org). This favors the development of bigger and more generic research software packages by consortia of partner institutions, which are intended to be used and relied upon also by scientists outside the laboratories that are actively involved in creating the software. This development calls for new workflows to provide researchers with adequate means of control over the exploding complexity of their software.

By way of illustration, in the past, the adoption of centralized version control systems, such as Subversion, enabled an enormous increase in productivity both for individual developers and small teams of researchers alike. However, in larger groups and under increasing pressure from more intense development pace, such systems can become a hurdle that inhibits agile processes due to branching/merging deficiencies, overhead from relying on the central server for most actions, and so on. The answer to this challenge lies in removing centralization overheads through the adoption of new distributed version control systems (DVCSs) that are designed around the notions of forking and branching. We hope that with the advent of DVCSs that are easier to use, the general acceptance of version control in scientific community will continue to steadily increase, further enhancing the productivity of the developers.

In this contribution, we would like to promote within the neuroinformatics community an upgrade in development practices of a similar spirit. *Continuous integration* (CI) (Beck, 1999) is a modern method of complexity control which is becoming increasingly popular in the software industry. A CI-based workflow ensures that changes made to the code base during the development process are fit for release *as they are implemented*, rather than immediately before the release. Distributing the integration of changes throughout the development cycle facilitates improvement of software quality and adherence to release schedules.

An essential prerequisite for CI to realize its full potential is a sufficient test coverage of the software in question. In order to appropriately test the software, one needs to break it down into a number of maximally self-contained units and check whether these units produce (previously known) correct outputs in response to the test data; such checks are called “unit tests.” In addition to that, every time a bug is identified and fixed in the software, extra tests (so-called “regression tests”) can be added to the test suite to ascertain that the problem is resolved for good and does not subsequently re-occur. Even though CI is already useful in absence of a test suite, for detecting breakages during the development process of projects written in compiled languages, its utility is boosted by orders of magnitude when applied to a code base with a robust test coverage.

Building upon the previous work on ensuring the correctness of NEST (Eppler et al., 2009), a collaboratively developed neuronal network simulator for large heterogeneous networks, we established a new CI-based development process. Our solution, described in section 2, is based on Jenkins, a popular CI server. It is scalable and open source: we share our designs and source code required to independently replicate a similar setup, in addition to providing CI as a service to a number of other projects.

The adoption of CI has resulted in sizeable improvements for all projects participating in our pilot implementation. In section 3.1 we describe how the basic features of CI allow the “health” of a code base to be measured, by providing valuable metrics which help to guide engineering efforts. In addition, we show how the CI system can indirectly contribute to other parts of the workflow, such as supporting a complex code merge (see section 3.2.1). The CI-based workflow in the NEST project not only allows us to detect a large number of potential problems that would previously have gone unnoticed until the integration phase, but also substantially decreases the turnaround time (TAT) before already identified issues are resolved (section 3.2.2). Other projects have benefited from advanced statistics collection (code coverage), static code analysis, regular multi-platform builds, etc. (see section 3.3).

In section 4 we identify several additional areas in which CI can be leveraged to aid development and quality assurance (QA) processes, and other approaches that can be used to manage the complexity of a growing code base. Finally, we argue for establishing a common platform that would provide similar services to neuroinformatics projects, thereby promoting the adoption of CI and thus raising the quality of research-oriented software in the field of neuroscience.

### 1.2. Continuous integration

**Figure 1:**
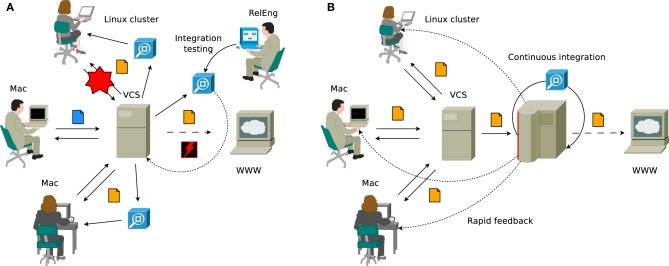
**(A)** Traditional workflow when quality control is applied to the project during the integration phase. The code is stored centrally in the version control system (VCS), but individual developers are relied upon to test their code regularly (the process of running the test suite on the checked out code is illustrated by the blue boxes, untested changes are denoted with blue file icon, whereas orange file icon indicates that the change set in question is tested). Changes are frequently introduced that cause breakages for some developers, but not for the others (exemplified by the red star, showing how checked in changes that work on Mac OS X broke the support for Linux platforms), which is often only discovered by release engineering. In the worst case, releases containing severe bugs might get shipped to the external users (represented by the red flash). **(B)** Agile continuous integration-based workflow, where a central server is set up to monitor the state of the source code, collect code metrics and perform automated testing. The changes are integrated into the main tree directly as they are being developed and releases are produced when all automated testing procedures pass. Regular testing is now being performed automatically by the continuous integration server in a standard setting (so no committed changes will remain untested) and developers are immediately notified in the case if committed changes have caused build breakages or test failures.

Let us consider a typical software engineering workflow in a project with multiple developers, illustrated in Figure 1A. Assume the group of scientists consists of a number of seasoned developers working on several platforms (for instance, Linux and Mac OS X). They are already employing a number of techniques that can thankfully be considered a standard in software development these days: the project's source code is hosted in a central version control repository and has a test suite that allows to validate its core functionality. Smaller changes are implemented directly into the main source tree, larger features are developed in branches that are merged into the main tree when new versions are released. Before the software is pushed to a public web server, a release engineer (most often this is a single person responsible for releases, but may also be a team) reviews smaller changes that made it into the trunk during the development cycle, merges outstanding changes from branches, runs the test suite and cuts the release. This process is known in the industry as *integration phase* and testing performed during this phase as *integration testing*.

Unfortunately, this workflow does not scale as the group of developers becomes larger and more geographically distributed, and the software itself evolves into a more complex solution consisting of a significantly greater number of lines of source code (SLoC) running on many subtly different platforms. Thus it becomes practically impossible for release managers to maintain awareness of the state of the code in different subsystems, where accumulating problems start calling for changes in overall design or simply require refactoring of the code to bring it up to the current standards (several such scenarios are shown in Figure 1). Typical symptoms of this state of affairs include constantly delayed releases, frequent breakages on some of the supported platforms and loss of control over the code base.

As the software gains more optional features, some developers effectively give up on regular testing before every commit (essentially committing untested code), since testing every single combination of features becomes prohibitively time consuming. Besides, even the most thorough developers do not have all of the supported platforms in standard configuration at their disposal and therefore, as illustrated, it often happens that while the test suite passes on one platform, the software is no longer operational on another (the latter is not a purely hypothetical speculation, but has in fact occurred on multiple occasions during NEST development). In the “best” case, these problems are identified later on in the development cycle when one of the developers is confronted with them during his research activities, but often they remain undiscovered until the integration phase and cause additional delays or, even worse, make it into release and are later reported by the external users.

To counter these accumulating problems, a new methodology called *CI* has been introduced by the agile development practitioners community, and is steadily gaining popularity in the industry. In a project that has adopted a CI-based workflow (illustrated in Figure 1B), quality control is *continuously* applied to the product as opposed to the traditional procedure of applying QA during the *integration* phase after completing all development. Essentially, CI is a way of decreasing the risks associated with integration by spreading required efforts over time, which helps not only to improve the quality of the software, but also to reduce the time taken to deliver it.

In practical terms, a typical solution consists of a dedicated master server, which monitors the central source code repository, controlling a build farm. As changes are detected, the system builds the code in a standardized environment, runs a battery of tests to validate it, collects useful metrics reflecting the state of the code, and provides the developers with immediate feedback. It is important to mention, however, that CI is not only about automating parts of the QA process, but can lead to a paradigm shift in the development practices when universally accepted in the team.

Stringent quality control is very important in the context of research-oriented applications in the domain of neuroinformatics, due to their emphasis on correctness, reproducibility and performance. Many projects, however, are restricted by the lack of manpower and funding to implement elaborate QA procedures. We assert that the NEST project is in a similar situation to many neuroinformatics projects: we cannot afford to keep developing *without* CI any longer, but at the same time, most “off the shelf” solutions are inaccessible to us due to our limited budget.

Here we present a blueprint for developing a low-cost, high functionality CI system, which meets the needs of the neuroinformatics community as proven by the conducted case studies. Technical implementation details follow in section 2, the effects of the CI on the development practices in NEST and the benefits for additional case studies of neuroinformatics projects are given in section 3 and finally, the conclusions and future directions are summarized in section 4.

## 2. Materials and methods

### 2.1. Supporting infrastructure: general directions

The scale of the supporting infrastructure for CI should generally match the level of complexity of the project at hand. Small projects, such as convenience libraries or focused stand-alone applications, especially written in an interpreted (and thereby to a large extent platform-independent) language like Python, do not generally require testing on many different operating systems and hardware platforms. Hence, an out-of-the-box instance of any popular CI server installed locally on the developer's workstation may be entirely appropriate. For large-scale projects, however, heterogeneous build farms consisting of dozens of servers, including those running various operating systems on rather unconventional non-x86 hardware (like POWER, SPARC, and ARM among others), are not uncommon.

Such large build farms, however, entail total costs of ownership (TCO) far beyond the price tag attached to the bare hardware (which in itself is often a challenge to fund), including administrative labor, hardware maintenance, network connectivity, power and cooling, rack space, and other concomitant overhead expenses. As most projects in neuroinformatics, NEST included, are run on tight budgets there is a natural desire to minimize these costs by increasing server density and maximizing hardware utilization.

Therefore, considering the scale of the NEST project, we adopted a compromise strategy: a single large x86 SMP server was dedicated to support the infrastructure for the CI system. Instead of deploying the CI server on bare metal and running builds directly on the master, we decided to virtualize all builders and machines running most auxiliary services through a Type 2 hypervisor (KVM) in order to remain flexible by isolating services from each other and also from the underlying hardware platform. In this setting, each particular service (e.g., web or application server, build slaves, etc.) is confined inside a virtual machine with a dedicated operating system installation running on top of emulated hardware, as if it were a separate physical server. This way individual services are disentangled to the largest possible extent, with the only remaining allowed interactions between them happening via the virtual network.

The obvious advantage of such a setup from the security standpoint lies in that if any individual service were to be compromised, the attacker would not necessarily gain full control over the whole system, which is important in the scenario when services are being offered to third parties that are only weakly trusted. In addition to that, virtualization provides both hardware abstraction and a higher amount of flexibility from the management point of view. Virtual machines can be freely migrated between physical nodes (provided that such nodes have required amounts of resources available) and individual services and/or their platforms (operating system and runtimes) can be upgraded, downgraded or reinstalled independently from each other.

Additionally, we invested significant effort in keeping all aspects of the infrastructure described in a declarative fashion (whereas the description itself is stored in a source control repository). Such an arrangement facilitates the distribution of administrative responsibilities among several (possibly geographically dispersed) operators on one hand, and on the other hand, allows every virtualized service to undergo virtual to physical (V2P) migration in the case that its resource requirements can no longer be satisfied by the host machine. For instance, if the need arose to test NEST builds systematically on POWER hardware and the emulation speed proved itself to be too slow for builds to be carried out at an acceptable rate on the current platform, an additional POWER-based blade could be installed in the rack and connected to the configuration management system in the same way as the virtualized guests.

Another advantage of keeping declarative definitions of all guest machines is that it is trivial to scale up the number of otherwise identical builders as needed. This is particularly important for matrix builds, when the CI system is set to try all possible combinations of build configuration parameters (in the specific case of NEST: by enabling or disabling the support for distributed computing through MPI, including or excluding models that make use of advanced integrators from GNU Scientific Library, etc.). As convenient and important as matrix builds are, unfortunately, the amount of possible individual configurations explodes with the number of build parameters. Therefore, the ability to increase the number of builders by simply duplicating already existing definitions is very advantageous, as in this way spare system resources can be used to keep build times within reasonable limits.

These two ground-laying approaches (virtualization and declarative configuration management) work well for the vast majority of hardware and operating systems currently available on the market. However, we need to mention two important caveats. First, it is often desirable to use Mac OS X slaves for CI, but the legal status of running virtualized copies of Mac OS X operating system by Apple on non-Apple hardware is ambiguous and depends on the jurisdiction. In this case, an exception for using a bare-metal system instead is warranted. Second, declarative configuration descriptions work best with platforms that incorporate advanced package management (i.e., most Linux distributions, various BSD flavors, Oracle Solaris, etc.). On platforms that adopt packaging only in a severely limited form, e.g., lacking package repositories or having an unpackaged base system (such as Microsoft Windows or Apple Mac OS X), installing and updating software through configuration management systems requires substantial extra effort. Systems administrators wishing to use these platforms should be aware of these additional shortcomings.

### 2.2. Supporting infrastructure: hardware and software

**Figure 2:**
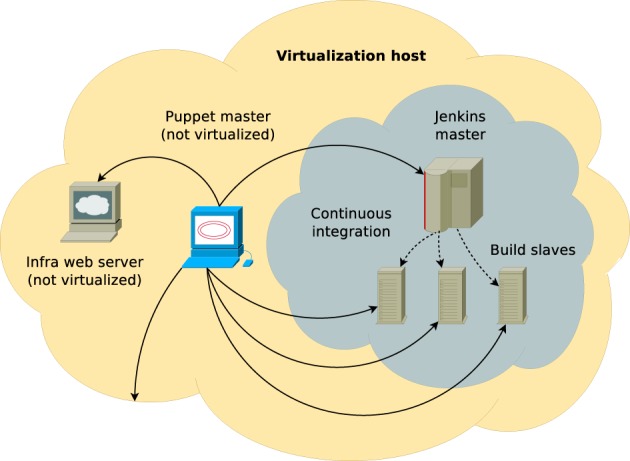
**Schematic diagram of NEST QA infrastructure.** The orange cloud represents the virtualization host (Dell PowerEdge R710 server) with a number of services running directly on top of the host operating system. The gray cloud corresponds to an isolated virtual network that connects virtualized build master and build slaves. All services are managed by non-virtualized Puppet master instance. There is bi-directional communication between build master and slaves, however, neither of them communicate directly with the host (only individual services, such as Puppet master and infrastructural web server are listening to the host's interface that is attached to the isolated virtual network).

The system is deployed on an off-the-shelf Dell PowerEdge R710 server (2U rack-mount format) featuring 2 x Intel Xeon X5680 CPUs (6 physical cores and 12 SMT threads each running at 3.4 GHz clock speed on average), 48 GB of RAM and 2 × 2 TB spinning disk drives in software RAID-1 configuration along with a single 100 GB solid state drive. The machine is running Red Hat Enterprise Linux 6.3 which is managed through Red Hat Network (RHN) online system management software and serves as a virtualization host as well as runs a non-virtualized Puppet 2.7 master (software versions are accurate at the time of writing). Guest machines are controlled via libvirt virtualization API, which also controls the firewall, e.g., sets up network address translation (NAT) and provides them with necessary core network services such as DHCP and DNS through dnsmasq. This scheme is illustrated in Figure 2.

Infrastructure description is implemented in Puppet (http://puppetlabs.com), stored in a publicly available git repository (see section 4.3) and fetched into the host machine manually as necessary. Puppet is the name of both an open source configuration management system implemented in Ruby and a declarative domain-specific language (DSL) based on Ruby and Puppet libraries, which can be used to write manifests for said configuration management system that contain descriptions of system resources and their states. In the following, the key concepts relating to declarative configuration management systems will be explained with application to Puppet for clarity, however, note that they are mostly independent of a particular software and can be readily translated to other systems such as Chef, etc.

The Puppet ecosystem comprises three inter-related main components: *Puppet master*, the server component that compiles operator-supplied manifests into system-specific catalogs containing information about resources and resource dependency, *Facter*, which is the utility that discovers relevant facts about the system, and *Puppet agent*, which is the client component that fetches appropriate catalogs from the master and applies them against target systems making use of facts. Additional supporting components include related software such as *Marionette Collective*, which enables mass actions to be performed remotely on Puppet-controlled hosts, and various control dashboards, lifecycle management systems such as *Foreman*, etc.

Once a new revision of manifests is downloaded to the server, Puppet master compiles catalogs that are relevant to each particular guest and distributes them upon next round of update requests. All guest machines are running Puppet agents which are installed at the provisioning stage, and by default they poll the master for updates every 30 min. This comes with an additional benefit of having a known state to be enforced on all machines: for instance, if any particular service crashes (or was stopped via manual intervention and not restarted thereafter due to operator error), it will be restored into the running state by Puppet agent upon the next execution.

It is interesting to note that since most machines do not have a state and associated non-volatile information, it does not make sense to back them up in this setting, because the whole setup can be reconstructed from the infrastructure definition which is stored in a version control repository. Since *git*, the system used to store the revisions, is a DVCS, each checkout contains the whole history of repository modifications and hence is a backup in itself. The few exceptions that do have states, such as the CI master server, can be dealt with on an individual basis.

A typical workflow when changing the configuration of the system then boils down to the following sequence of actions: describe the updates in form of Puppet resources, commit updated manifests into the git repository, fetch them into the server, perform a *dry-run* —a special mode of operation of the Puppet agent, when necessary changes to the system are displayed, but not actually applied—and finally push them into production. Puppet also supports *environments* which make it possible to implement staged workflows when only servers in the test lab or specific subsets of production servers receive updated manifests, but the added complexity of such a solution outweighs its advantages in our particular case.

Provisioning of new machines plays a crucial role in the overall infrastructure maintenance. In order to make it a predictable and reproducible process, we use kickstarting to automate the deployment of the hosts, be it a physical or a virtual machine. This is a mechanism available for Red Hat derived systems, including the community-supported Fedora distribution. Kickstart files containing various directives to the operating system installer are automatically generated by Puppet from templates using the information about the guests that is already exposed otherwise following the DRY (do not repeat yourself) principle.

### 2.3. Continuous integration server

There are several common CI system designs to chose from: a master/slave model, reporting server model, and a hybrid of both. In order to avoid limiting the setup to a particular build system stack (e.g., CMake and CDash) and to keep it as versatile as possible, we selected a hybrid model where both server can send commands to the build slaves and the slaves can periodically report to the server [see Brown and Wilson (2011), Chapter 9. “Continuous Integration” for the details, the book is freely available at http://www.aosabook.org/en/].

We chose the long-term support release of Jenkins (LTS) for its stability and infrequent upgrade cycle (every 1–2 months, regular releases are pushed out every week) as the CI solution implementing this paradigm, and one virtual machine was dedicated to run the master server. The web application is served through its bundled servlet container; the option of deploying through a 3rd party application server was not considered, since no other Java applications were to be deployed and so using an external container would not offer any advantages at this scale. The Jenkins master was set up to control its slaves via secure shell connections (SSH) and via Java Network Launching Protocol (JNLP) slave agents where SSH is unavailable (Windows).

Jenkins allows the functionality and appearance of the basic system to be extended by installing additional plugins. The project provides plugin developers with an extensive and stable API, which makes it possible to customize almost every aspect of the system without modifying its core. The importance of having such an API should not be underestimated, because this makes it possible to upgrade the system easily without having to re-implement site-specific customizations. Additionally, Jenkins developers operate a centralized plugin update server which greatly simplifies the task of keeping track of the new versions of extra plugins. Users can also set up their own trusted update environment should the need arise.

In our case, we supplemented the standard Jenkins setup with several non-core plugins, most notably a set of plugins for build log and static code analysis/metric collection (Static Analysis Utilities, Jenkins Cobertura Plugin, Warnings Plug-in), as well as extra plugins for most popular version control systems (VCSs) (Jenkins Mercurial Plugin, Git Plugin). The community around Jenkins is generally very open to external contributions. The changes to core plugins that we developed during the work described here were deemed to be useful outside the context of our specific site. Following an independent code review, they were accepted upstream, thus removing the need for us to maintain them on our own.

Thanks to the advanced permission system built into Jenkins and strong isolation achieved by virtualizing the builders, we were able to extend the service to several other software packages. We provided Jenkins accounts to users external to the NEST Initiative, with sufficient permissions to modify the build jobs for their respective projects without interfering with other parts of the system. This made it possible for them to enjoy the benefits of having a centrally managed CI solution without incurring additional costs.

We adopted the following policy with respect to (re-)creating suitable build environments on Jenkins slave machines: all necessary configuration changes are to be made through Puppet right after kickstarting. This also implies that all necessary software packages and libraries should be installed system-wide via the integrated package management facilities. On one hand this can be seen as an inconvenience, because some most recent libraries and programs might not be available directly from the operating system repositories, so they need to be backported or packaged manually and fed into the local infrastructural repositories. On the other hand, this approach ensures perfect automated reproducibility of the setup, also for the downstream users that might wish to rebuild from source on their own machines in an identical way.

## 3. Results

### 3.1. General overview

The core benefit provided by the adoption of CI-based development practices is the ability to offset the risks associated with the integration phase by distributing required efforts over time, i.e., integrating new changes into the product as they are being developed as well as performing statistics collection and quality control on the go. This increases the quality of the software while decreasing the time to market for new features and bug fixes. In addition to monitoring VCSs, scheduling builds and running the test suites, one of the most important functions of a CI setup is to extract and collect information about the builds. Developers thereby have access to indicators that show the status of the latest build and a convenient interface to the historical data.

**Figure 3:**
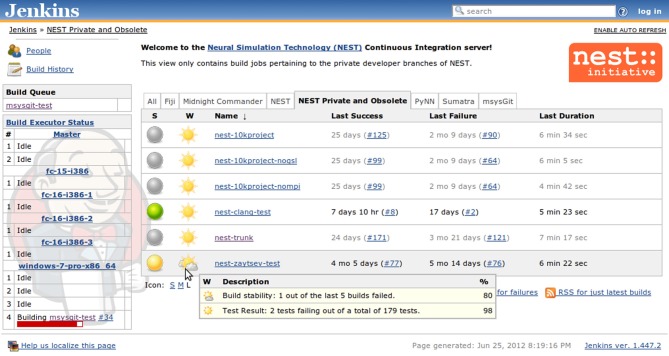
**Jenkins build job summary that aggregates information about the jobs registered with a particular view.** The table in the middle shows for each job the build status (green balls for successful and stable builds, yellow for unstable builds, red for failed builds [not shown], and gray for disabled jobs), a weather report that includes several indicators, such as stability, test results, code coverage, and static analysis (not illustrated), etc., as well as links to the individual pages of the last successful and last failed builds. On the left-hand side there is an additional table that shows the build queue and the current status of the available builders.

All the kinds of information that are collected by Jenkins are summarized in Table 1. Jenkins provides a very intuitive and ergonomic web interface to access this data (Figure 3) which can be supplemented by e-mail notifications and/or other interfaces and notification schemes provided by the plugins, among others CLI interface, and IRC and Jabber notifications. The availability of a one-stop information point for build and test results as well as code quality metrics has had a significant positive effect on our development workflow, as we discuss in greater detail in section 3.2.

**Table 1:** **Summary of data collected by Jenkins build jobs and presented in the on-line web interface or stored in Jenkins home directory for further processing**.

**Information source**	**Collected data**	**Explanation**
Version control system	Committed changes	Most jobs are set to monitor version control systems for new commits, hence, when such commits are identified, authors and commit messages are recorded for further reference. For instance, if a build fails, the commit author can be notified directly
Source code	Static analysis	Jenkins can be made to run various static code analysis tools and parse their output. Examples of such tools include SLoC counters and style checkers such as duplicate/dead code analyzers and bug/vulnerability discovery tools; historical information can be used to plot trends and/or compute build “health” indicators
Build logs	Compiler warnings	Compilers often emit warnings to draw users' attention to potential problems with the code; upon completion of a build, the log can be scanned for warnings emitted by known compilers (GCC, LLVM, etc.)
	Test results	Many test suites produce reports in machine-readable format, most notably JUnit; if the format is supported by Jenkins, test results can be collected and parsed automatically upon every build
	Coverage metrics	Some test suites (or compilers) include instrumentation for tracking code paths that have been exercised during the test suite run; if the report format is supported by Jenkins, this information can be collected to asses the quality of the test suite
Build results	Build duration	The amount of time that the build takes is recorded and can be used as a rough performance indicator
	Build status	The exit codes of the build scripts are recorded and analyzed; if non-zero, the build is marked as failed. Additionally, build can be marked as unstable (or failed) if the compilation succeeds, but there are test failures or other problems such as an excessive amount of warnings, or code coverage drops below a user-defined threshold, etc.
	Weather report	The build status history for a particular job is represented as a “weather report,” which shows how many builds have failed in succession and thereby provides means to asses the “health” of the code base

The same web interface can be used to configure most aspects of build jobs and the Jenkins system as a whole, which helps to distribute administrative responsibilities among developers. This functionality is, of course, protected by password and therefore not visible to the public. In order to grant the rights to configure their own build jobs to external developers from associated projects we use the *Project-based Matrix Authorization Strategy*, the most flexible built-in access control and authorization strategy available in Jenkins. It allows us to assign different sets of permissions to individual accounts or groups of accounts globally, and then specify fine-grained settings per build job if needed.

#### 3.1.1. Build log analysis

One of the most frequently used features of a CI system is undoubtedly the analysis of build logs; not being a part of the core Jenkins distribution, it is provided by Dr. Ullrich Hafner's *Static Code Analysis Plug-ins* suite (https://wiki.jenkins-ci.org/display/JENKINS/Static+Code+Analysis+Plug-ins). This collection of plugins offers a robust build log analysis data collection and visualization framework. In the following, we describe a specific part of this suite, the *Compiler Warnings Plug-in*, in greater detail, but other analogous collectors such as *Checkstyle, DRY, FindBugs*, and so on are also available.

**Figure 4:**
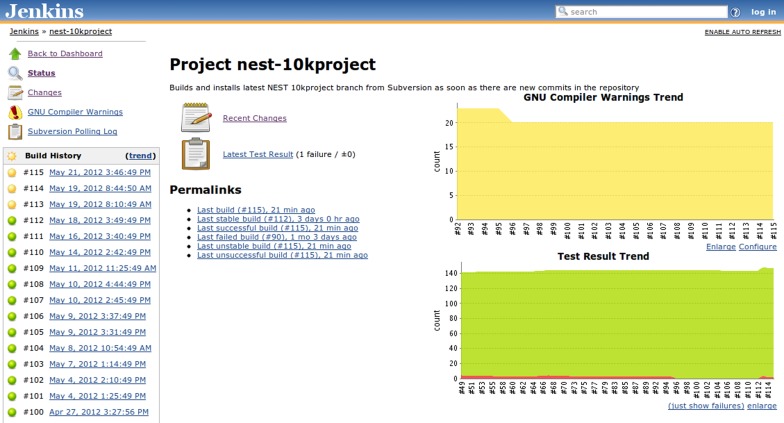
**Jenkins build job view featuring the build job set up specifically for the “10K Project” branch mentioned in the main text (section 3.2.1).** The build history bar on the left side shows the outcomes of the recent builds and provides links to the detailed information, i.e., which changes triggered the build, detailed build log, etc. Additionally two trend graphs are present on the right side: compiler warnings and test result trends. The compiler warnings graph shows the evolution of the number of warnings over time, but does not discriminate particular kinds of warnings. The test result graph shows the proportion of failing tests (red area stacked under the green one) over the past 65 builds.

Most developers are aware of the importance of writing clean code that does not cause compilers to emit warnings, however, in practice this goal is difficult to achieve. As large projects are compiling, huge amounts of verbose build system output scroll by and intermittently appearing new warnings are very hard to detect, especially if some warnings are in fact generated by inclusion of headers from external libraries and should not be taken into account. One way around this problem is the “-Werror” GCC flag, which instructs the compiler to treat warnings as errors and abort the compilation as soon as a warning is encountered. This option is not always applicable, as in the case mentioned above, when some warnings are beyond the control of the project. A less uncompromising, but nevertheless useful tool for making warnings stand out in *Autotools-based* projects is the AM_SILENT_RULES macro, which makes the output of the build system substantially less verbose, but still informative and hence all kinds of warnings and error messages become significantly easier to spot. Other build systems also implement similar facilities. However, this approach still requires humans to track warnings, which makes it less attractive. Conversely, the Jenkins warning collection as illustrated in Figure 4 does not require any additional efforts on the part of the developer. Warnings are collected automatically and plotted as a trend, new and fixed warnings are recorded and can be easily examined. Finally, the administrator can set a threshold amount of warnings to mark the build as failed whenever it is exceeded, analogous to the “-Werror” feature of GCC.

#### 3.1.2. Matrix builds

The more powerful and feature-rich a project becomes, the harder it is to test it properly. It often happens that some additional functionality is added that depends on an optional library, however, it is important to make sure that the software can still be compiled and remain functional in its basic configuration. Alternatively, sometimes certain functionality is provided by several competing packages (i.e., SQL-compatible database engines or different MPI implementations) and it is necessary to test the software against each of these packages to be confident that possible subtle implementation details do not affect the results (Gronenschild et al., 2012). Finally, software may be deployed on a variety of architectures (the most common example are x86-based 32-bit and 64-bit CPUs) and implicit low-level assumptions such as the sizes of the primitive types, may lead to compilation errors or even incorrect computation results on some of the platforms.

Clearly, it is impossible to put the responsibility for this kind of testing on the shoulders of the developers every time they make a change to the code base. Even with just three binary choices, the number of possible combinations amounts to eight, which rapidly adds up to an unacceptable waiting time even if a single build takes a relatively short amount of time. In addition, the developer has to track which combinations he has already tried, and this also becomes impractical very quickly.

**Figure 5:**
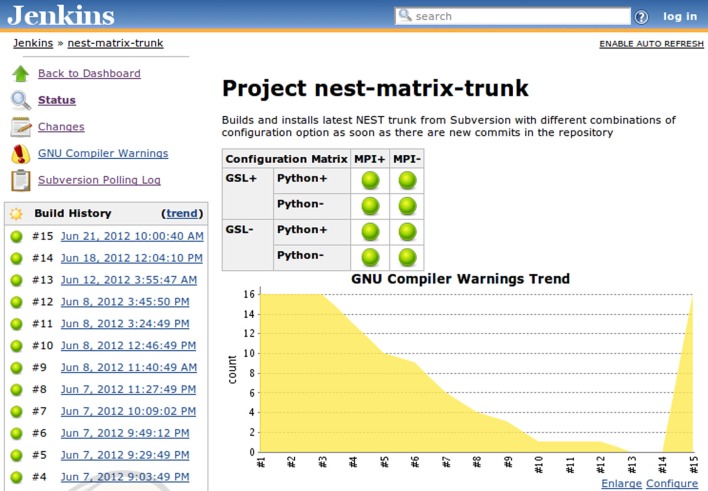
**Matrix build set up for the main branch of NEST.** Every time a change is committed to the repository, all major configurations are built, installed and tested to catch regressions in the functionality in a timely manner.

This is where the matrix builds come in: the administrator only needs to define the “axes” of parameters by entering all possible values thereof, or by tagging groups of slaves with particular labels. Examples of parameter axes might include build options, such as “--with-mpi” or “--without-mpi,” or the architectures or operating systems of the build slaves. Using this information, Jenkins will generate all possible combinations of parameters and intelligently schedule builds to optimize the utilization of the slaves. Build results are presented in a summary table as illustrated on Figure 5 which gives easy access to the detailed information about each individual build.

Unfortunately, with the number of builds grows the waiting time until all of them are completed. One strategy to avoid the dramatic increase in response time without putting more strain on the budget is to define *canary* and *touchstone* builds. In the former case, the CI server does not execute dependent build jobs if the canary build (that is supposed to be reasonably fast) fails, thus sparing resources for other useful builds. In the latter case, several manually specified (most important) builds from the matrix are always executed first in order to provide developers with rapid feedback on build failures; remaining builds are carried out as free slots become available.

### 3.2. Improvements to the nest development workflow

We set up our CI system, built as described in section 2, to regularly poll the VCS hosting the main official NEST source tree. Whenever changes are detected, the latest source code is downloaded from the VCS to a build executor machine and the following actions are performed: the build system is bootstrapped, NEST is built and installed in a temporary location and a test suite run is initiated. The builds are performed on a volatile file system (tmpfs), since compilation and linking is generally an I/O-intensive operation and hence doing it completely in-RAM dramatically speeds up the process. NEST is built in a variety of configurations to make sure that code changes do not inadvertently cause regressions for some less often used combinations of features (Figure 5). Test reports (in JUnit format) as well as build logs are recorded and archived on Jenkins master node for further analysis.

**Figure 6:**
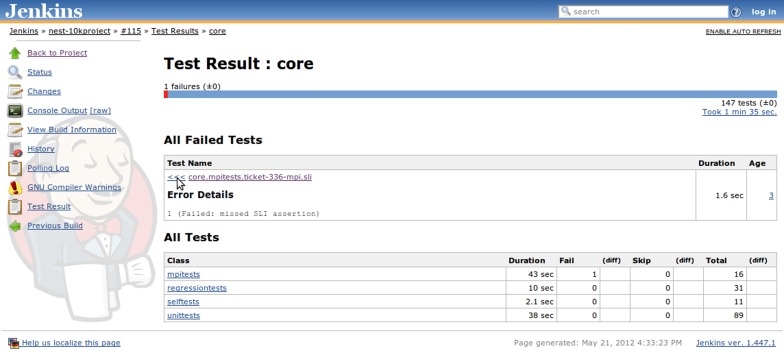
**Jenkins detailed test report view also featuring links to other details pages, such as build log (console output), information on which changes triggered the build, historical trends for test results, and warning analysis, etc.** For failed tests, one can immediately check out the return code or exception raised (depending upon which information is provided by the JUnit generator) or browse to the details page which provides a full backtrace. Tests are aggregated in packages and for each package the total duration, total number of tests, as well as the amount of failed and skipped tests are reported in the summary table.

Build logs are then subjected to an automated search for GCC/LD or LLVM Clang warnings, which are recorded and used to plot trends, compute “weather” indicators and create table-based presentation with breakdowns according to affected source files, etc. Same goes for test suite reports, which are also used to plot trends and create tabular test result representations that enable developers to easily check for the backtraces of failing tests, asses the average amount of time required to run particular test sets and so on (see Figures 4 and 6). Beyond the much improved overview of our code base obtained through these measures, the introduction of the CI-based workflow has very positively affected the development practices within the NEST project, illustrated in sections 3.2.1 and 3.2.2.

#### 3.2.1. Merge workflow

One of the most important advantages of the new workflow that we experienced is related to merges. Large changes integrating substantial new functionality that can potentially affect vast amounts of code are usually carried out in branches, which allows developers to experiment with new simulation techniques without jeopardizing the stability of the main code base. However, the more radical the changes, the longer it takes to implement them, especially with limited resources at hand. Therefore, as the development of the main code base goes on, branches diverge further and further from the trunk, making the ultimate merge of the branch back into the trunk an increasingly painful and complicated process.

We conducted one such major merge (Kunkel et al., 2012) shortly after putting our CI server into production and opted to use it as an opportunity to experiment with CI-based workflows. To this end, we created a number of dedicated build jobs for the branch in question, subsequently replaced by a single matrix build. All of the core developers got together in the same room and the CI status page was projected on a wall screen as the merge was being worked on. This way the developers were provided with instantaneous feedback with regards to the current status of the branch and as soon as issues arose, the problem spots were quickly identified and fixed. Moreover, the system was collecting and displaying statistics on the numbers of (yet) failing tests after integrating every new block of changes, which made it easy to maintain a good understanding of which areas still require priority developer attention despite the rapidly changing situation. Overall, this new setting tremendously improved our productivity and relieved a lot of stress usually associated with such merges.

#### 3.2.2. Turnaround time for resolving broken builds

An indirect, but important, advantage of CI is related to the following human factor: the earlier the developer is notified of an issue with the patch that was just committed, the easier it is for him or her to associate this regression with specific changes in code that could have caused the problem and fix it. If the regression is discovered much later or by a different developer, it usually takes much more time and effort to find out the root cause of the problem and eliminate it.

In absence of a CI server, obviously, no such statistics have been regularly collected and hence without concrete numbers most evidence for apparent improvements with the introduction of CI would forcibly remain anecdotal. Therefore, in order to obtain a quantitative assessment of how much the development workflow of NEST has been enhanced by implementing CI, we conducted the following experiment: we examined every single revision of the NEST source code that is compatible with the GCC 4.3 compiler, covering a period of around 4 years (r7732–r9712). For each revision we made a clean checkout, bootstrapped the build system, configured NEST with default parameters, built and installed the executable, and ran the test suite. Whenever any of these steps failed, the whole build was regarded as failed. Individual test failures as such were not considered, the build was marked as failed only if the test suite failed to launch. The builds were carried out in parallel on a local HPC grid in order to obtain the results within reasonable amount of time.

**Figure 7:**
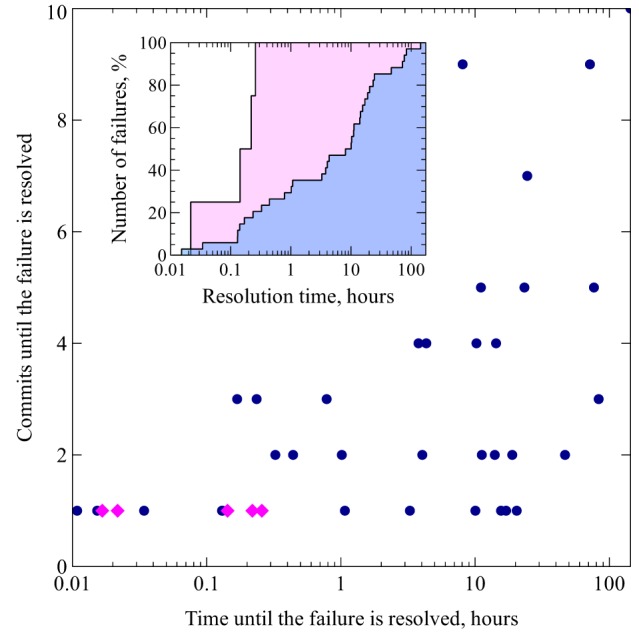
**Scatter plot illustrating the relationship between the number of commits and the amount time that it took to fix a build failure.** Failures that occurred between September, 2008 and September, 2011 are marked with blue circles, failures that happened since October, 2011 (when instant build failure notifications were enabled thanks to the CI system) are marked with magenta diamonds; time values are represented on a logarithmic scale, while the numbers of commits are equally spaced. The inset shows the same data in the form of a cumulative histogram: the proportion of build failures on the vertical axis is plotted against the time until the failure is resolved on the horizontal axis (logarithmic scale). The blue area corresponds to the blue circles on the scatter plot (failures that occurred before the introduction of CI notifications) and the pink area corresponds to the magenta diamonds (failures that happened thereafter).

As can be seen on Figure 7, before the introduction of CI the time to fix failures was very variable, and some issues remained unsolved for days. The cloud spreads toward the upper right corner of the plot, which is consistent with the expectation that severe breakages would require more time and take more attempts to get fixed. In contrast, since the developers started getting instant notifications of build failures from the CI system, no failure remained unresolved for more than half an hour (see Figure 7, inset).

### 3.3. Benefits for other associated projects

Besides NEST proper, we provide hosted CI services to a number of other projects. Our contribution was recognized as a very useful and important one by all of the participants. Even in case of Fiji, where a CI system already creates nightly builds, we were able to identify new bugs. This was because our setup is managed externally to Fiji project and some common assumptions of the developers that apply to their own setting do not hold for our system. Additionally, we are providing Windows builds for every commit for Fiji project, which were previously not available.

In the case of the PyNN and Sumatra projects, we were able to leverage the power of code coverage metrics. These projects are implemented in Python, which can be easily instrumented to collect code coverage information without any changes to the project test suite, thanks to an add-on “coverage.py” library by Ned Batchelder. Using coverage metrics, the problem spots in the test suites could be easily identified and scheduled to be better covered with additional tests.

For Midnight Commander (mc), in addition to a regular job that builds development branches and enforces consistent code indentation policy, we set up a static analysis job using Clang Static Analyzer, which is a perfect match since this project mostly consists of pure C code. As a result, a large number of possible issues of different severity have been identified, ranging from ignored return values to null pointer dereferences and logic mistakes. Leveraging this positive experience, we intend to apply similar static analysis techniques to NEST code base in the future.

### 3.4. Cost estimates

The infrastructure necessary to implement CI may be provisioned in multiple ways. For instance, one may opt to use a cloud provider alone (such as Amazon, Linode, and others) or in conjunction with a “CI-in-a-Cloud” offering from companies providing CI as a hosted service, like CloudBees, ShiningPanda, etc. These offers are quite attractive from the prospective of scientific software developers, for whom infrastructure maintenance is typically not the major focus, because setup, administration and hardware maintenance tasks are all abstracted away by the cloud provider.

Since many such providers have a free starter plan, and are especially welcoming to open source software projects, this could be a very interesting opportunity for the developers that only want to try this new workflow out without making large investments or time-consuming commitments upfront, when the scale of the benefits is not yet clear. CloudBees, in particular, offer BuildHive service (https://buildhive.cloudbees.com) as a part of their DEV@cloud platform for free to open-source projects; this service integrates with GitHub (https://github.com, the social coding platform also available free of charge for hosting public code repositories) and can be used to not only automatically build and test newly committed code, but also validate pull requests coming from project contributors, all with minimal effort. A similar community service exists under the name of Travis CI (http://travis-ci.org): it also integrates with GitHub and provides support for a number of different development platforms, including Python, Ruby, C/C++, and others.

It should be borne in mind, however, that convenient as they are, cloud services can quickly become rather costly. Careful consideration must be therefore given to determining the point at which purchasing a dedicated infrastructure would be a more economical solution. Additionally, instant scalability, the biggest advantage of cloud-based services, does not really play a role in the case of providing CI and so would not justify increased costs. We performed a rough estimate of the needs of the NEST project for the month of June 2012 and ended up with a figure of at least 60 h of production building and testing per month (3 projects × 15 builds each × 8 configurations × 10 min per build), excluding research builds, static analysis, etc. According to the current CloudBees DEV@cloud pricing, this would cost us around $250 (~€200) per year and we would be limited by two parallel executors at every point in time (which means that matrix builds would take a substantially larger amount of time to complete).

At the same time, the price tag for a 1U infrastructure server substantially better specced than Amazon's “m1.small” instance provided in this package is only around €500, even without any special offers or academic discounts. Although this implies additional systems administration overheads, the upside of having one's own machine lies in the ability to install any latest software without being limited by the realms of a hosted offering. This may be especially important for C/C++ projects that depend upon a number of third-party libraries, which all need to be deployed to the build slaves in a particular fashion.

Another attractive option for a project with a severely limited budget to procure build machines at virtually zero additional cost is to recycle hardware. University computer centers and institutes which manage their own IT infrastructure regularly determine that a given machine is no longer economically viable to be used for its present function and that they would rather use the rack space for something else. Such redundant machines can be readily repurposed by a small lab or project that would not be able to afford to buy the hardware new. To give a real-world example, a part of the BCF HPC Grid (one rack of IBM eServer 326 machines featuring 4 GB of RAM and 2 × AMD Opteron CPUs each) is now being retired. Although these compute nodes are in perfect working condition, they have become too expensive to operate as a part of a grid by modern standards, especially considering their power consumption and concomitant need for cooling, manageability, operating system support, etc.

These outdated machines, however, can make a perfect set of builders for CI infrastructure similar to ours. In terms of administration, they can be centrally managed through Puppet just as the virtual machines in our setting, since Puppet is completely network transparent; in fact, managing networked computers is its primary purpose. Regarding power efficiency, it is important to consider that only the Jenkins master needs to remain online for continued operation, whereas the slaves can be kept offline most of the time and only started up on-demand using Wake-on-LAN (WOL) by Jenkins sending magic packets to the required machines as the builds are being scheduled. The only remaining concern is finding an appropriate space to accommodate the recycled hardware.

For the presently described research project, we deployed our own virtualization-based CI infrastructure as described in section 2. The rack space and cooling facilities are maintained by Bernstein Center Freiburg, whereas the network infrastructure is provided by the Computing Center of the University of Freiburg. The administrative labor is divided among the developers of NEST and associated projects, and a systems administrator at the Bernstein Center. The administrator is responsible for keeping core virtualization infrastructure as described in section 2 up and running, including software updates (approximately 10 h per month), whereas the maintenance of the build jobs is delegated to the developers thanks to the authorization and role distribution system mentioned in section 3.1. Of course, the developers can also reduce the administrative workload by sending patches for the publicly available declarative description of the infrastructure (see section 4.3), but are not required to do so. We estimate that for someone already familiar with virtualization and declarative configuration management, it would take approximately 20–30 h to reproduce the solution presented here on the basis of our documentation and Puppet code. For someone without such prior experience, additional time would be necessary to become familiar with the concepts and tools.

In our case, the costs of the resulting system are dominated by the price of the hardware (€8400 at the time of purchase in August 2010). However, the system described in the present paper is also used as a research prototype and its capacity is largely excessive for smaller projects or projects requiring less flexibility. In such cases, an appropriate CI system could be set up on a much less expensive machine (such as a typical 1U infrastructure server mentioned above). At the time of writing a comparable system-based, for instance, on SuperMicro X9DRW-iF barebone would cost roughly €4200 without taking into account any special offers or discounts usually available to academic customers.

## 4. Discussion

### 4.1. Summary

**Figure 8:**
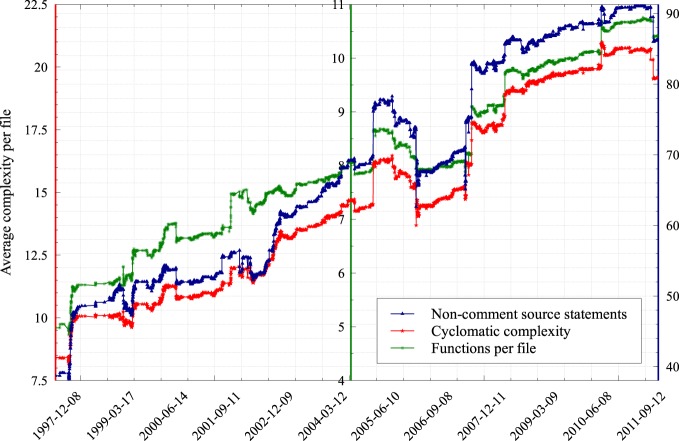
**The evolution in time of the average complexity per file of the NEST simulator code base.** In this experiment we applied three different industry-standard techniques for estimating the complexity of a code base to every single revision recorded in the NEST version control repository. Cyclomatic complexity is a metric that measures the number of linearly independent paths through the source code (McCabe, 1976); the definition of “non-commenting source statement” is available at the homepage of CppNcss (http://cppncss.sourceforge.net/reference.html), the software that we used to collect this metric; the number of functions per file was measured according to the definition of function pertaining to the C++ standard.

NEST is a collaborative project to maintain and further develop an efficient simulation technology for large heterogeneous networks of neurons that addresses the concerns for reproducibility and correctness. From the inception of the project, the complexity of the code has been growing steadily. Figure 8 shows the average complexity per file of the NEST code base according to three different metrics. The metrics we selected are highly correlated, but interestingly, in our case, the most primitive one, being the average count of functions per file, turned out to be a good predictor for the two others, which are much harder to compute. This is due to the average cyclomatic complexity and the number of non-comment source statements per function remaining reasonably stable over time (data not shown).

All three metrics agree that the complexity of the code has almost tripled over the course of 15 years. NEST can no longer be characterized as a piece of software that is being authored by a few collaborating researchers from the same laboratory. Instead it is a product that is developed by a large number of geographically dispersed scientists who are working on the code simultaneously. It therefore represents an excellent test case of a neuroinformatics project having a strong need for new solutions to manage the complexity of jointly maintaining such a large code base.

In this paper, we present the combination of software and techniques required to implement a new CI-based software development workflow, highlight its advantages for sustainable development of a code base and report the quantitative and qualitative improvements to our code development practice. It is important to emphasize that we did not simply install an “off the shelf” system on our hardware and document this process, but rather created a scalable open source design for deploying same class of systems and are offering our code, documentation and experiences to the neuroinformatics community.

By virtue of using virtualization to partition the host system, it is already flexible enough that we were able to provide CI as a service to other projects, whereas by employing a declarative configuration management solution, we made it possible to readily re-use our code by projects having similar needs. In fact, we have already received several external contributions to our tools from individuals working in totally different areas, such as the hosting industry, which underlines the importance of sharing code to promote its re-use across fields and domains.

### 4.2. Future work

As extensions to our current work, we have identified a number of other practices and instruments to investigate further. Many of these are common in the software industry, but have yet to establish themselves in the scientific software development community. Here we address two areas that enable the development of better quality software for less effort: processes and tools. We discuss these potential improvements from the point of view of the NEST project, but they can be easily applied to other neuroinformatics projects.

#### 4.2.1. Development process

***4.2.1.1. Continuous delivery.*** A natural extension to CI-based workflow is a practice of *continuous delivery*, where *any* build is considered as a potential release candidate as long it satisfies the QA requirements. Here the decision to release or not is taken on the basis of objective criteria, such as the amount of passing tests (if the code base is sufficiently well-covered, the percentage of passing tests after each commit becomes a reliable measure of the projects condition) and other health indicators, as opposed to deadlines or inherently subjective feelings of the release engineers. Therefore, changes in release strategy are the next logical step after successful implementation of a CI-based workflow. By adding few bits of automation, releases can be safely cut directly from the CI system, which will remove error-prone manual steps from the release process and save valuable time to the developers.

Since one of the important goals for us is to provide the access to the latest features and bugfixes as soon as they are available, two release feeds can be made available: a “stable” feed, where releases are cherry-picked by release managers from the CI builds which received the most positive feedback, taking mainly their feature-completeness into account, and an “unstable” feed that has all the latest commits and passes all tests, but potentially containing features that are highly experimental in nature, incompletely implemented and tested, or have interfaces that are subject to change.

***4.2.1.2. Code review.*** Another important practice that may dramatically improve the quality of code submissions is a centralized code review solution. In recent years, several code review tools, such as Gerrit (http://code.google.com/p/gerrit/), have been gaining momentum, among others, thanks to provisions for tight integration with CI workflows and DVCSs. Gerrit in particular can act as a “smart” Git server that generates web-based code review interface from pushes to the repository and triggers CI to automatically test the incoming changes.

Effectively, this provides the developers with an easy way to access the CI build farm and testing facilities *before* the change is actually integrated (in addition to a convenient interface for soliciting reviews from their colleagues and line-by-line commenting of the changes), without the extra complexity of having to maintain developers' own build jobs in the CI system, additional access credentials, etc. In turn, the ability to run full integration tests earlier (“in the background”) as the code is being developed results in higher quality submissions. It also encourages the developers to break down their changes into smaller chunks that can be easily reviewed and tested, and reduces the load on their development computers.

***4.2.1.3. Performance monitoring.*** An interesting opportunity that is created by the implementation of the automated testing of every new change in a stable well-defined environment is the systematic performance assessment of the software. Previously, this was next to impossible due to the amount of labor required and differences among developer machines that make it hard to compare the benchmark results, etc.

We plan to add an additional component to the NEST test suite that consists of tests that are designed to measure the required CPU time and amount of memory needed to simulate several key scenarios; the infrastructure to plot performance trends from JUnit reports already exists in Jenkins. Note that unlike the other modules of the test suite, the performance measurement module is not primarily intended to be run by the developers as part of their development practice, but only in well-defined benchmarking environments. Similar solutions have already been employed by a few open source projects, such as PyPy (http://speed.pypy.org) and have proven to be of great utility to keep track of how the performance of the code base evolves in time, attribute regressions to changes in particular components and have them fixed quickly. This approach can also supplement memory modeling-based techniques (Kunkel et al., 2012) to guide development.

#### 4.2.2. Development tools

***4.2.2.1. Static and dynamic analysis.*** Given a large enough developer/tester and user base, most bugs are quickly identified and fixed [otherwise known as Linus's Law (Raymond, 1999)], and, likewise, perfect test coverage prevents serious issues from sneaking into releases. Unfortunately, neither of these maxims generally applies to scientific software projects, limited by the amount of the available resources and short of external testers/users. Therefore, instead of solely relying on testing, one should be actively looking to confront bugs and fix them, before they result in a failed simulation or corrupted data analysis. Taking advantage of the ability to easily automate systematic collection and plotting of virtually any metric offered by the CI setup, we would like to capitalize on static (Bessey et al., 2010) and dynamic code analysis techniques.

For instance, we recently introduced weekly LLVM Clang builds, which produce a set of concise and informative warnings that do not overlap with those of GCC. Clang Static Analyzer, unlike many other tools, especially the free ones (e.g., cppcheck, http://cppcheck.sourceforge.net) uses the power of LLVM internal representation (IR) to identify execution patterns that are often causes of bugs. Unfortunately, it does not yet properly support C++ and, therefore, we are investigating alternative solutions, including commercial tools (such as PVS-Studio, http://www.viva64.com/en/pvs-studio/).

Another interesting option would be to research the integration with Valgrind (http://valgrind.org), a framework that provides thread error detectors, instrumentation to track down memory errors, etc., but at this point, its reports are hard to interpret without manual assessment by a human, so additional exploration is required to make it practical.

***4.2.2.2. Testing and QA.*** In order to strengthen our QA process, we have already introduced matrix builds described in section 3.1.2 to cover the most important branches of NEST. Next we will extend them to run the same sets of builds on different CPU architectures: some differences in NEST behaviors on 32-bit and 64-bit machines have already been identified in the test setting during the research phase of this project, which drew our attention to code issues that would not have been observable using a single architecture. Automating this kind of testing with CI, as well as extending it to other popular architectures, such as ARM, POWER, etc., is one of our priorities to ensure perfect reproducibility of simulations across platforms.

However, as prevention is at least as important as diagnosis, we feel that improving the quality of NEST test suite (Eppler et al., 2009) itself is another obvious and important long-term goal. In order to achieve it, we need to evolve the test suite in two main directions: first, we need to make sure that the test suite covers the whole simulator's code base reasonably well, leaving no untested “black holes” behind, and, second, that running it should not only prove that *well-behaved code passes the tests*, but also that *incorrect code fails the tests*. Ideally, the assessment of how close are we to fulfill these two requirements should be unambiguously quantifiable, rather than based on personal opinions or intuitions of the developers.

The first aim can be reached by adopting the metric known as “code coverage,” as reported in section 3.3. For that, we would need to introduce instrumentation into the NEST engine in order to be able to collect code coverage information from all NEST layers (C++, SLI, PyNEST) and feed it into Jenkins. This would allow us to identify the weakest parts of the test suite and refine it, guided by well-defined objectives and appropriately chosen metrics.

The second issue can be addressed by adding support for fuzz testing (Sutton et al., 2007) and mutation testing (Offutt, 1994) to NEST. Fuzzing means providing random unexpected or invalid data to the input of the program in order to reveal unhandled exceptions such as crashes, failing assertions, etc. The concept of mutation testing involves exposing the source code or compiled byte-code to a mutagen, which will randomly introduce specific changes to operators or opcodes, for instance, replace comparison operations with their negation (i.e., “>” → “≤”) or cause other changes designed to mimic typical programming mistakes. If the test suite is properly designed, such changes should entail test failures. In the case that such failures do not occur, this signifies that the essential logic is not being properly covered. This is a likely outcome, as the tests are designed by humans, who are inherently cognitively biased by having prior expectations on *how* a particular function might fail. Random mutations and fuzzed input data remove this subjectivity and therefore have the potential to expose test coverage problems. However, such testing is a time and resource consuming operation and therefore an ideal subject for automation via CI.

### 4.3. Outlook

The fundamental aim of NEST Initiative, as a major collaborative project in neuroinformatics, is to provide a modern and efficient platform for carrying out point neuron simulations larger and larger in scale, which integrates well with other simulation software (Djurfeldt et al., 2010). On one hand, this includes visualization solutions and models for population signals reconstructed from single spikes such as EEG/BOLD, etc., and on the other hand, detailed neuronal simulators that feed information into NEST. In order to remain a solid foundation to build upon, NEST must offer usable low-level and high-level interfaces to all this functionality. Following from the requirements above, both the size and the complexity of the code base of NEST will certainly keep growing in the future (Figure 8). The same conclusion can be drawn for many neuroinformatics projects.

The goal of good scientific software development should not simply be to create an application that is reliable in the sense of compiling and running without crashing. To support high quality science, it must also be reliable in terms of accuracy and generate results that can be independently reproduced and verified. In the specific case of simulators, a major aspect is the requirement that the simulation of a model should accurately reproduce the mathematical model it is based on, and that the presence of approximations that would potentially cause deviations be made explicit to the user.

The ideal way to achieve this goal is, of course, to end up with a formal proof of correctness of the program. Unfortunately, with currently available tools, this is hardly practical even for small and primitive codes, let alone large scientific software applications. It is only being carried out (at huge expense) in areas where correctness is literally a matter of life and death, such as aeronautics or energy. Consequently, our only remaining option is to try to contain this complexity. One way to go about it is to adopt new complexity control technologies as soon as they are sufficiently mature; i.e., they have reached the point at which it is practical to implement them in the setting of research-oriented software projects run on tight budget by developers who didn't major in applied software engineering (Wilson, 2006).

In the present paper we highlight one such method focusing on software development workflows, *CI*. We believe that it has become ripe and are hoping for its widespread adoption, as has previously taken place with source control and unit tests, and present our case study along with the designs and source code such that our solution can be easily reproduced. Nevertheless, we realize that not all projects, especially smaller ones, can immediately afford to build such a system. A superficial survey of the INCF Software Center (http://software.incf.org) revealed that out of more than 300 projects, less than 10 explicitly mention that they are using some form of CI, e.g., Slicer (CDash), NiPy and Topographica (buildbot). This suggests that either the awareness of CI is low in the neuroinformatics community, or that the hurdles to implement it have been too high.

Therefore, as an experiment, we opted to provide CI as a hosted service to a certain number of other neuroinformatics projects. The feedback so far was overwhelmingly positive and the use of the described new workflow has benefited all of the participants. However, this solution does not scale due to the limited resources and manpower that we were able to dedicate to this cause. Consequently, we conclude that the availability of a centrally administered *CI as a service* offer is crucial for further improving the quality of neuroinformatics software, the increasing complexity of which constantly challenges the developers to remain in control over their code bases. As a result of the current study, the Simulation Lab Neuroscience at the Jülich Research Center, in collaboration with the INCF, plans to establish a scaled-up version of the solution presented here as a service to the neuroinformatics community.

CI is of course not the only method of complexity control that can be considered in the neuroinformatics context. Among other noteworthy approaches, we can mention the drift toward functional programming, where functions are designed such that when executed they do not alter any global state of the running software (or, in other words, do not have side effects). Wider adoption of this technique would enable programmers to write better tests, since *pure* functions can be covered much more easily. Additionally, it would simplify the verification of the crucial parts of the software with automatic theorem provers and (more realistically) boost the development of increasingly robust static analysis tools of all kinds. At the same time, advanced instrumentation, such as coverage metrics and mutation testing, will help to approach test suites in a principled fashion, guided by informative measurements, while workflows and process part will be covered by agile methodologies, CI and continuous delivery.

We conclude that it is important to keep in mind that CI is only one piece of the puzzle (Wilson et al., 2012), albeit an important one. Given the ever increasing importance of software for the advancement of neuroscientific research, the problem of complexity management will require a proactive approach toward identifying, adapting and exploiting future techniques and technologies emerging in the software industry.

### Conflict of interest statement

The authors declare that the research was conducted in the absence of any commercial or financial relationships that could be construed as a potential conflict of interest.

## References

[B1] Beck K. (1999). Extreme Programming Explained: Embrace Change. 1st Edn Reading, MA: Addison-Wesley Professional

[B2] Bessey A. Block K. Chelf B. Chou A. Fulton B. Hallem S. (2010). A few billion lines of code later: using static analysis to find bugs in the real world. Commun. ACM 53, 66–75

[B3] Brown A. Wilson G. (2011). The Architecture Of Open Source Applications. Mountain View, CA: lulu.com

[B4] Djurfeldt M. Hjorth J. Eppler J. M. Dudani N. Helias M. Potjans T. C. (2010). Run-time interoperability between neuronal network simulators based on the MUSIC framework. Neuroinformatics 8, 43–60 10.1007/s12021-010-9064-z 20195795 PMC2846392

[B5] Eppler J. M. Kupper R. Plesser H. E. Diesmann M. (2009). A testsuite for a neural simulation engine, in Proceedings of the 2nd INCF Congress of Neuroinformatics (Pilsen, Czech Republic).

[B6] Gronenschild H. B. M. Ed. Habets P. Jacobs H. I. L. Mengelers R. Rozendaal N. van Os J. (2012). The effects of FreeSurfer version, workstation type, and Macintosh operating system version on anatomical volume and cortical thickness measurements. PLoS ONE 7:e38234 10.1371/journal.pone.0038234 22675527 PMC3365894

[B7] Kunkel S. Potjans T. C. Eppler J. M. Plesser H. E. Morrison A. Diesmann M. (2012). Meeting the memory challenges of brain-scale network simulation. Front. Neuroinform. 5:35 10.3389/fninf.2011.00035 22291636 PMC3264885

[B8] McCabe T. (1976). A complexity measure. IEEE T. Soft. Eng. 2, 308–320

[B9] Offutt A. (1994). A practical system for mutation testing: help for the common programmer, in Test Conference, 1994. Proceedings, International, (IEEE) (Washington, DC), 824–830

[B10] Raymond E. S. (1999). The Cathedral and the Bazaar – Musings on Linux and Open Source by an Accidental Revolutionary. Beijing, China: O'Reilly

[B11] Sutton M. Greene A. Amini P. (2007). Fuzzing: Brute Force Vulnerability Discovery. Upper Saddle River, NJ: Addison-Wesley Professional

[B12] Wilson G. (2006). Where's the real bottleneck in scientific computing? Scientists would do well to pick up some tools widely used in the software industry. Am Sci. 94, 5

[B13] Wilson G. Aruliah D. A. Brown C. T. Chue Hong N. P. Davis M. Guy R. T. (2012). Best practices for scientific computing. arXiv:1210.0530v3. Available online at: http://arxiv.org/abs/1210.0530v3 10.1371/journal.pbio.1001745 PMC3886731 24415924

